# Diverse Head-to-Tail Sequences in the Circular Genome of Human Bocavirus Genotype 1 among Children with Acute Respiratory Infections Implied the Switch of Template Chain in the Rolling-Circle Replication Model

**DOI:** 10.3390/pathogens13090757

**Published:** 2024-09-03

**Authors:** Kexiang Zhang, Ri De, Yanpeng Xu, Zhenzhi Han, Runan Zhu, Yu Sun, Liping Jia, Dongmei Chen, Yutong Zhou, Qi Guo, Yao Yao, Shuang Liu, Dong Qu, Yuan Qian, Linqing Zhao

**Affiliations:** 1 Laboratory of Virology, Beijing Key Laboratory of Etiology of Viral Diseases in Children, Capital Institute of Pediatrics, Beijing 100020, China; 18800198237@163.com (K.Z.); graceride@163.com (R.D.); yanpxbj@163.com (Y.X.); hansir8@sina.com (Z.H.); runanzhu@163.com (R.Z.); sunyu780312@163.com (Y.S.); im_jiaping@126.com (L.J.); dongmei_c@126.com (D.C.); 18601399785@163.com (Y.Z.); g7siete1220@163.com (Q.G.); xinongyaoyao@163.com (Y.Y.); yqianbjc@263.net (Y.Q.); 2 Graduate School of Peking Union Medical College, Beijing 100730, China; 3 Department of Intensive Care Unit, Affiliated Children’s Hospital, Capital Institute of Pediatrics, Beijing 100020, China; shuangliu1105@163.com (S.L.); qudong2012@126.com (D.Q.)

**Keywords:** children, human bocavirus genotype 1, head-to-tail sequence, diverse, rolling-circle replication model

## Abstract

Head-to-tail sequences have been reported in human bocavirus (HBoV) 1-4. To reveal their features and functions, HBoV DNA was screened among respiratory specimens from pediatric patients with an acute respiratory infection (ARI) between April 2020 and December 2022, followed by HBoV genotyping. Head-to-tail sequences were detected using nested PCR, TA cloning, and Sanger sequencing, and these findings were confirmed by mNGS and amplicon sequencing. The secondary structure was predicted using the Mfold web server. The results indicated that head-to-tail sequences were detected in 42 specimens through TA cloning from 351 specimens positive for HBoV1 DNA, yielding 92 sequences into 32 types and 2 categories. Additionally, head-to-tail sequences were detected in 16 specimens by amplicon sequencing, yielding 60 sequences categorized into 23 types. The 374nt type, detected in 13 specimens, contains variants 374a and 374b, which differ in the unpaired loop regions of the palindrome or complementary reverse sequences, implying a switch of template chains during the replication process. The mNGS results in three specimens confirmed the presence of circular genome in copies below 1%. In conclusion, head-to-tail sequences of HBoV1 were common in children with ARI and were highly diverse in length and sequences. The variants may be generated by the switch of the template chain in the rolling-circle replication model.

## 1. Introduction

Human bocavirus (HBoV) was first discovered and identified in 2005 from respiratory specimens by random PCR amplification, large-scale gene sequencing, and bioinformatics [1]. This virus was subsequently named HBoV1 to distinguish it from HBoV2 to HBoV4, which were found in the stool specimens of children with acute gastroenteritis from 2009 to 2010 [2,3,4]. It has been confirmed that HBoV 1 is a genuine pathogen responsible for acute respiratory tract infections in pediatric patients, as determined by nucleic acid, antigen, and serology tests [5]. In several worldwide clinical studies, HBoV1 has been identified as one of the most common respiratory viruses in young children with respiratory tract infections. HBoV1 causes a variety of respiratory diseases in children, including common cold, acute otitis media, pneumonia, bronchiolitis, and asthma exacerbation. With the accumulation of data, more severe, life-threatening, and even fatal respiratory HBoV1 infections have been reported worldwide [6]. However, the pathogenic relevance of HBoV1 has long been questioned for two main reasons. First, polymerase chain reaction (PCR)-based techniques limit the ability of clinicians to establish causality. Second, HBoV1 is frequently identified simultaneously with other respiratory viruses [7]. Furthermore, viral persistence hinders the diagnosis of acute HBoV infection, making the detection of viral DNA in nasal swabs highly sensitive, but not very specific. It has been suggested that no test alone is sufficient in all instances for accurate HBoV1 diagnosis in severe infections. Molecular assays and the serology of paired serum samples should be combined and considered together with other laboratory data, clinical features, and the time of symptom onset [8].

HBoV1 is a linear single-strand DNA (ssDNA) virus, with 95% negative-sense strand DNA and 5% positive-sense strand DNA. It was classified to the Bocaparvovirus genus of the Parvoviridae family [9]. The length of the reported genome sequences of HBoV1 is diverse, including prototype sequences St1 in 5217 base pair (bp) (GenBank No. DQ000495) and St2 in 5299 bp (GenBank No. DQ000496), BJ3722 in 5299 bp reported in our laboratory (GenBank No. DQ988933) [10], and the longest one in 5671 bp (GenBank No. OL519570) from Xuzhou, China. The HBoV1 genome sequence contains three major open reading frames (ORFs), ORF1 encoding nonstructural proteins (NSs), ORF2 encoding a specific nuclear phosphoprotein (NP1), and ORF3 encoding viral capsid proteins (VP1-3s) [11,12]. In the 5543 bp genome sequence of HBoV1 (GenBank No. JQ923422), there is a 3′left-end hairpin (3′LEH) with an imperfect “rabbit-ear-type” palindromic hairpin of 140 nucleotides (nt), and a 5′right-end hairpin (5′REH) contains a perfect palindromic structure of 200nt [13]. The 3′LEH is critical for junction resolution, generating the ssDNA genome from the replicative-form DNA for encapsulation into capsids [13,14,15]. A minimal replication origin (OriR) located from nucleotides 5357 to 5402 contains the NS1 binding elements (NSBEs) (nt5366-5373) and the nicking site (nt5381-5390). The OriR serves as a template for HBoV1 DNA replication initiation.

The replication pattern of parvoviruses has been reported as the rolling hairpin model on the basis of the head-to-head or tail-to-tail intermediates detected [16,17,18]. However, head-to-tail sequences, instead of head-to-head or tail-to-tail intermediates, were detected in clinical specimens positive for HBoV1-4 [19,20,21,22]. Especially, the circular genome sequence HBoV2-C1 (GenBank No. JX257046) was detected from fecal sample BJQ435 in our laboratory [22]. Three hetero-recombinant HBoV2-C1 genome clones suggested that structures retaining in the head-to-tail sequences are important for HBoV2 DNA replication and virus assembly [23]. The head-to-tail structures suggest a rolling circle replication model for HBoVs, in which the ends of a linear DNA molecule are connected to form a circular structure and effectively undergo multiple rounds of replication by keeping the template strand stable during the replication process [24]. However, the features and functions of these head-to-tail sequences of HBoVs, especially in HBoV1, a genuine pathogen for ARI in children, have not been understood.

To reveal the characteristics and functions of the head-to-tail sequences of HBoV1, especially their relation to rolling-circle replication, respiratory specimens collected from pediatric patients with acute respiratory infections (ARIs) between April 2020 and December 2022 were screened for HBoV1 DNA. The head-to-tail sequences were amplified by nested PCR and cloned into a TA vector for Sanger sequencing. Some of these sequences were further deep sequenced by amplicon sequencing, while others were validated using Meta-Genomic Next-Generation Sequencing (mNGS). Based on the results of the head-to-tail sequences, the rolling-circle replication model was proposed.

## 2. Materials and Methods

### 2.1. Clinical Specimens

Clinical specimens, including throat swabs, nasopharyngeal swabs (NSs), nasopharyngeal aspirates (NPAs), and bronchoalveolar lavage fluids (BLFs), were collected from pediatric patients with ARI who visited the Affiliated Children’s Hospital, Capital Institute of Pediatrics between April 2020 and December 2022 for respiratory pathogen screening using a capillary electrophoresis-based multiplex PCR (CEMP)-compatible assay (Ningbo HEALTH Gene Technologies Ltd., Ningbo, China) [25]. These clinical specimens were diluted in a viral collection buffer containing protein stabilizer, ampicillin, kanamycin, and antifungal antibiotic at PH 7.0 (Yocon Biotech, Beijing Co., Ltd., Beijing, China). Upon arrival at the laboratory, all clinical specimens were centrifuged at 500× *g* for 10 min. The supernatant was divided into two parts; one part was used for nucleic acid extraction, and the other was stored at −80 °C for later use.

### 2.2. Nucleic Acid Extraction

Nucleic acid was extracted from 140 µL of each specimen using the QIAamp Viral RNA Mini Kit (250) (Qiagen, Hilden, Germany), according to the manufacturer’s instructions.

### 2.3. CEMP Assay for Multiple-Pathogen Screening

In the CEMP assay kit for multiplex PCRs, components such as deoxynucleotide triphosphates (dNTPs), MgCl_2_, and buffer are included. Nucleic acid extractions from clinical specimens were amplified and then subjected to capillary electrophoresis on a GeXP capillary electrophoresis system (Sciex, Concord, ON, Canada), according to the manufacturer’s instructions. The CEMP assay includes 15 different pairs of primers—thirteen for target pathogens, one for human DNA, and one for human RNA—with one primer of each pair labeled with fluorescein. Each pair of primers (a forward primer and a reverse primer) amplifies one target fragment. Different amplification products have different lengths. A fluorescently labeled size standard was added to the product. These samples were analyzed by a capillary electrophoresis analyzer. Smaller fragments move quickly, and larger fragments move slowly. By comparison to the migration time of the size standard, the various lengths of the PCR product fragments were determined and specific pathogens were detected, as well as human DNA and human RNA: influenza virus A (Flu A) 105nt (2009H1N1 163.3nt, H3N2 244.9nt), Flu B 212.7nt, human adenovirus (AdV) 110.2/113.9nt (representing different subtypes), HBoV 121.6nt, human rhinovirus (HRV) 129.6nt, human parainfluenza virus (PIV) 181.6nt, chlamydia (Ch) genus 190.5 nt, human metapneumovirus (hMPV) 202.8nt, mycoplasma pneumoniae (Mp) 217nt, human coronavirus (HCoV) 265.1nt, and human respiratory syncytial virus (RSV) 280.3nt [25].

### 2.4. Genotyping of HBoVs by PCR

For HBoV-positive specimens detected by CEMP assay, primers HBoV-c1 (5′-CTTYGAAGAYCTCAGACC-3′) and HBoV-c2 (5′-TKGAKCCAATAATKCCAC-3′) were used to amplify the 690-nt fragment at the boundary of NP1 and VP1 genes, followed by sequencing and phylogenetic analysis, as described previously [26].

### 2.5. Amplification, Cloning, and Sanger Sequencing of Head-to-Tail Sequences and Assembly of the HBoV1 Circular Genome

The head-to-tail sequences of HBoV1 were amplified by a nested PCR from specimens positive for HBoV1. The primers were designed according to the non-coding region sequence of HBoV1 (JQ923422), including the outer primers HBoV-F1-Tail (+) and HBoV-1-R1-Head (−) for the first-round PCR, and the inner primers HBoV-1-F2-Tail (+) and HBoV-1-R2-Head (−) for the second-round PCR (Table 1). These primers were synthesized by Shanghai Invitrogen Biotechnology Co., Ltd. (Shanghai, China) and purified by PAGE. Amplicons of 300–500 bp from the second-round PCR were purified and ligated with the pGEM-T Easy vector (TransGen Biotech, Beijing, China) in 4 °C overnight for TA cloning, then the recombinant DNA was transferred into competent *E. coli* DH5α cells. Up to five clones were selected from each plate and sequenced to obtain the head-to-tail sequences. The linear genome sequences of HBoV1, including genes NS1, NP1, VP1, and the 3′-noncoding regions, were amplified and sequenced from specimens positive for head-to-tail sequences, according to the method developed in our laboratory [9]. Then, the genome sequences of HBoV1 were ligated together with the head-to-tail sequences to obtain the circular genomes.

### 2.6. Amplicon Sequencing of Nested PCR Amplification Products

From each specimen, a total of 0.2 μg of DNA from the nested PCR amplification products was utilized as input material for DNA library preparation. The DNA fragments were end-polished, A-tailed, and ligated with the full-length adapter, followed by further PCR amplification. Then the PCR products were purified using the AMPure XP system (Beckman Coulter, Beverly, MA, USA), and the library quality was assessed using the Agilent 5400 system (Agilent, Santa Clara, CA, USA) and quantified by QPCR (1.5 nM) [27,28]. The qualified libraries were pooled and sequenced on Illumina platforms using the PE250 strategy at Novogene Bioinformatics Technology Co., Ltd. (Beijing, China), according to the required effective library concentration and data amount.

After sequencing and data quality control, forward and reverse sequences were assembled into consensus sequences using Paired-End reAd mergeR (https://cme.h-its.org/exelixis/web/software/pear/doc.html, accessed on 19 June 2024). The obtained consensus sequences were statistically analyzed for abundance based on sequence length. The base analysis performed on sequences of a fixed length with abundance higher than 3% determined the types of head-to-tail sequences.

### 2.7. Sequence Analysis of the Head-to-Tail Sequence

Sequences from pGEM-T Easy recombinant vectors and amplicon sequencing were compared with GenBank sequences using NCBI–BLAST to identify the presence of head-to-tail sequences and determine their sequence characteristics. The Mfold web server (http://www.unafold.org/, accessed on 5 July 2024) was utilized to predict the secondary structure of head-to-tail sequences.

### 2.8. Meta-Genomic Next-Generation Sequencing (mNGS) of Circular Genomes

Selected specimens, both with and without head-to-tail sequences, underwent genomic sequencing using mNGS technology on the Novaseq 6000 platform from Illumina (San Diego, CA, USA). A 2 × 150 cycles paired-end sequencing protocol was applied to each library, targeting a data yield of 10 GB. After high-throughput data quality control, full-length genomic sequences were de novo assembled using Megahit (version 1.2.9) and SPAdes (version 3.13.1) software. The sequences were annotated with nucleotide BLAST, setting an E-value threshold of 10-5. Viral genome sequence data were processed using MEGA (version 7.0) and BioEdit (version 7.0.9.0). Clean reads were mapped to the reference genome (GenBank No. LC756667) with Bowtie2 v2.3.4.3 [29]. The mappings were manually checked and merged from de novo assembled contigs and consensus sequences to calculate sequencing depth and coverage of the assembled full-length genomes. Gene annotations were analyzed using VAPiD v1.6.6. IGV (version 2.15.2) was employed to count the abundance of various representative reads and estimate the relative abundance of the circular genome. Reads containing both partial 3′ LEH and 5′ REH were identified as head-to-tail reads. The relative abundance of the HBoV1 circular genome in a specimen was defined as the percentage of head-to-tail reads among all HBoV1 reads.

## 3. Results

### 3.1. Pathogen Screening by the CEMP Assay

Between April 2020 and December 2022, 7787 clinical specimens were collected from 7506 children, with a male–female ratio of 1.38:1 and an average age of 3.32 ± 3.09 years. Among these, 351 specimens (4.51%, 351/7787) were positive for HBoV DNA, collected from 342 children with a male–female ratio of 2.51:1 and an average age of 2.26 ± 1.53 years. All were confirmed as HBoV1 through genotyping PCR and sequence analysis. Of the 351 positive specimens, 238 (67.81%, 238/351) were only positive for HBoV1, while 113 (32.19%, 113/351) were co-infected with other pathogens. The most common co-pathogen was HRV, found in 75 (66.37%) of the 113 co-infected cases, followed by PIV (19.47%, 22/113), hMPV (7.96%, 9/113), RSV (6.19%, 7/113), AdV (5.31%, 6/113), MP (3.54%, 4/113), HCoV (2.65%, 3/113), Flu (0.88%, 1/113) and Ch (0.88%, 1/113).

### 3.2. The Head-to-Tail Sequences of HBoV1 by TA Cloning and Sanger Sequencing

**Figure 1:**
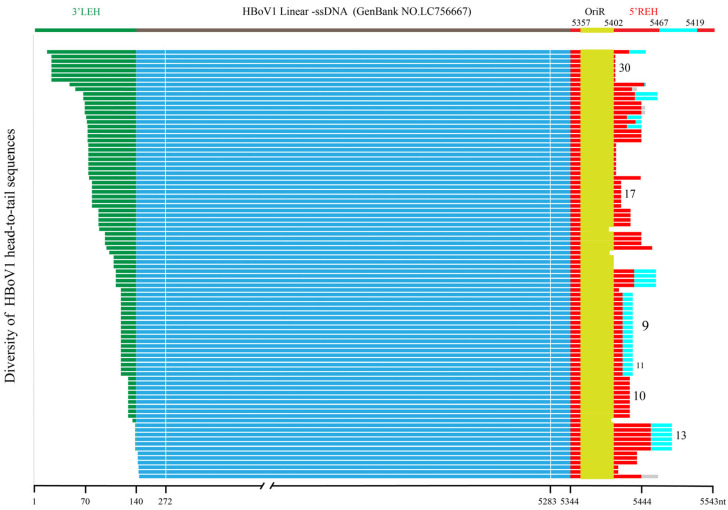
Mapping of the full-length sequences of 92 HBoV1 circular genomes. The circular genomes were compared to the reference genome sequence LC756667, with 3′LEH highlighted in green, 5′REH in red, OriR in yellow, and linker sequences in cyan, which are present in partial head-to-tail sequences. The linear genome sequences of HBoV1 are shown in blue. Different line segments under the linear genome of LC756667 were used to represent the 92 circular genomes, which were categorized into 32 types based on the alignment results of highly divergent head-to-tail sequences. The white line indicated the location of the nested second-round primer of PCR for detecting head-to-tail sequence. The number on the right represents the type of head-to-tail sequences. 3′LEH: 3′left-end hairpin, 5′REH: 5′right-end hairpin, OriR: minimal replication origin at the right-end hairpin.

Among the 351 specimens positive for HBoV1, 42 were positive for the HBoV1 head-to-tail sequence (11.97%, 42 out of 351), and a total of 92 head-to-tail sequences were obtained through TA cloning. By assembling linear genomic amplicons with head-to-tail sequences, the full-length circular genomes of HBoV1 were obtained with lengths ranging from 5231nt to 5409nt, which were then categorized into 32 distinct types based on the diversity of their head-to-tail sequences (Figure 1). The most common circular genome sequences were grouped into Type 9, with a length of 5298nt, accounting for 15.22% of the total (14 out of 92 sequences). This was followed by Type 10, also 5298nt in length, representing 9.78% of the sequences (9/92). Other notable clusters included Type 13 (5317nt, 6.52%, 6/92), Type 17 (5336nt, 6.52%, 6/92), and Type 30 (5384nt, 6.52%, 6/92). Out of the 42 specimens, 31 (73.81%) contained one type of circular genome sequence, and 11 contained more than one type of circular genome sequence, including 8 (19.05%) with two distinct types, 2 (4.76%) with three types, and 1 (2.38%) with four types.

Based on the head-to-tail sequences, these 32 types of circular genomes can be classified into 2 categories: The first category, which lacks linker sequences, includes 19 types comprising 53 sequences (53/92, 57.61%), while the second category, which contains 2–31nt linker sequences, includes 13 types comprising 39 sequences (39/92, 42.39%). Most of these linker sequences (35/39, 89.74%) shared high homology with other parts of the 5′REH sequences of LC756667, primarily within the range of nt5467–5519, while the remaining four linker sequences (4/39, 10.26%) shared homology with unknown sequences (Table 2). In particular, the linker sequences of type 9 and type 11 are identical to each other, with the only difference being one nucleotide at position 127 of the 3′LEH of JQ 923422: Type 9 has an adenine (A), and Type 11 has a guanine (G).

By linear alignment and secondary structure prediction of those head-to-tail sequences, the tail sequences were the most conserved, followed by the head sequences, while the linker sequences in the middle were the most diverse. There were 89 head-to-tail sequences (89/92, 96.74%) containing a complete OriR, and 3 (3/92, 3.26%) containing only NSBEs and nicking sites. All these sequences form stem–loop through 5′REH, regardless of whether there is a linker sequence.

### 3.3. The Head-to-Tail Sequences of HBoV1 by Amplicon Sequencing

A total of 16 specimens were selected for amplicon sequencing, including 5 randomly selected from those containing only one type of circular genome, and 11 containing more than one type of circular genomes. The sequencing run produced 5.77 G of raw data, with 5.67 G of clean data obtained through the filtering process. The number of clean reads obtained per specimen ranged from 767,936 to 1,765,456, with an average of 1,418,607.

**Figure 2:**
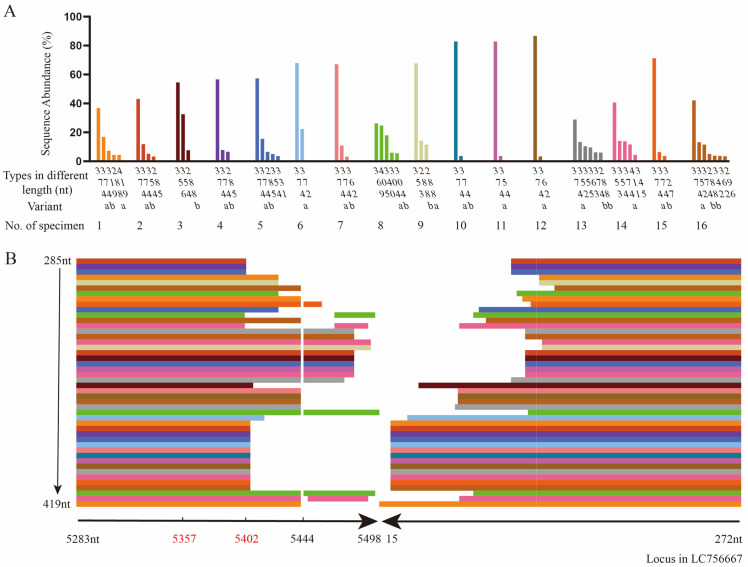
The diverse head-to-tail sequences determined by amplicon sequencing compared to the reference sequence LC756667. (**A**) The types in different lengths (nt) and variants (a and b) in different sequences of the diverse head-to-tail sequences with different abundances (%) identified in specimen No. 1–16. Clusters in different colors are specimen No. 1–16 selected correspondingly. (**B**) The alignment results of types in different lengths from 285nt to 419nt of head-to-tail sequences from specimen No. 1–16 compared to the reference sequence LC756667 from 5283nt–5498nt and 15nt–272nt.

A total of 60 head-to-tail sequences, with abundance over 3% and length from 285nt to 419nt, were produced from the 16 selected specimens and classified into 23 types of different lengths. Among the 23 types, the 374nt head-to-tail sequences, found in 13 specimens, were the most frequently detected with an average abundance of 63.77% (variance σ^2^ = 0.04), followed by the 288nt sequences, detected in 5 specimens, with an average abundance of 9.81% (variance σ^2^ = 0.007) (Figure 2A).

By ligating the 3′- and 5′-end sequences of LC756667 together and using it as the reference sequence, the sequence analysis shown in Figure 2B indicated that all tail sequences from 5283nt to 5402 or 5498nt contain OriR (5357nt–5402nt), while the head sequences were from 272nt to 140 or 15nt. There were 12 sequences with linker sequences and 48 without. High homology was found between these 48 linker sequences and the ligated LC756667. All head-to-tail sequences containing the 5444nt, the unpaired base at the end of 5′ palindromic sequence, are different at the loci from the reference sequence.

By aligning these 60 head-to-tail sequences with LC756667, the results indicated that one type of head-to-tail sequence with the same length, as determined by amplicon sequencing, could show different sequences (Table 3). For the 374nt type, there were variants 374a and 374b; both were detected in nine specimens with an average ratio of 7.24:1, while only 374a was detected in an additional four specimens. For the 288nt type, there were variants 288a and 288b; only 288a was detected in one specimen, only 288b was detected in three specimens, and both 288a and 288b were found in one specimen (Figure 2A). The differences between the variants of one type focused on the middle of the head-to-tail sequences.

**Figure 3:**
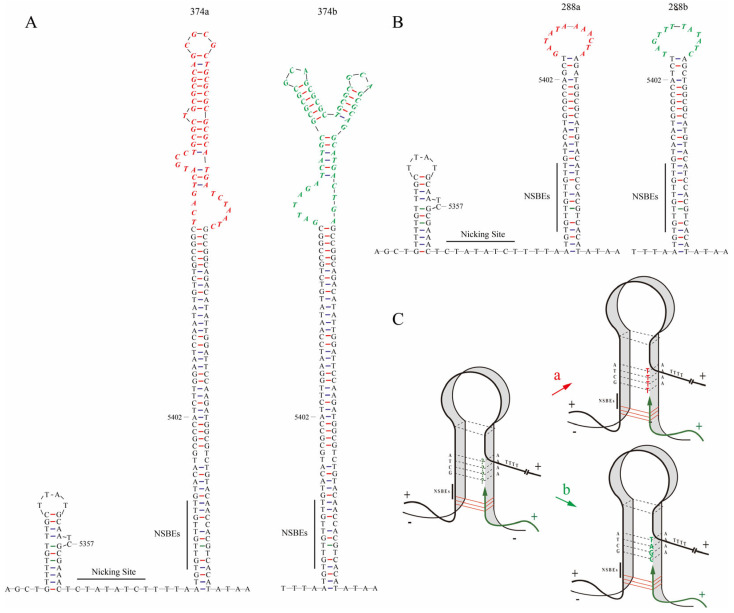
The predicted secondary structures of different variants in two common types, 374nt and 288nt, by Mfold web server, and a schematic diagram of the switch of template chains during replication. (**A**) The predicted secondary structure of 374a and 374b in type 374nt. (**B**) The predicted secondary structure of 288a and 288b in type 288nt. The head-to-tail sequences contain a stem–loop structure and an OriR region that is essential for replication. This region, spanning from nucleotide 5357 to 5402, includes the NSBEs and the nicking site. (**C**) The variants a and b of the same type may be generated from the switch of template chains during replication, with “a” indicating the complementary chain used as the template chain and “b” indicating the palindromic chain used as the template chain. The complimentary reverse sequences in variants a and b of the same type are shown in red and green colors, respectively.

The secondary structures of all head-to-tail sequences were predicated by MFold. The results indicated that a special stem–loop structure was formed at the junction of the head and tail sequences (Figure 3A,B). The differences between the variants of one type occurred in the unpaired loop regions of the palindrome sequences and were in complementary reverse, which implied the switch of template chain in the process of replication (Figure 3C). The unpaired loop of 374a was 5′-TCAGTCATGCCTGCGCTG CGCGCAGCGCGCTGCGCGCGCGCATGATCTAATC-3′, and the unpaired loop of 374b was 5′-GATTAGATCATGCGCGCGCGCAGCGCGCTGCGCGCAGCGCAGGCATGAC TGA-3′. The unpaired loop of 288a was 5′-GATATAAAACTA-3′, and 288b was 5′-TAG TTT TATATC-3′. They are complementary reverse sequences, respectively.

### 3.4. Meta-Genomic Next-Generation Sequencing (mNGS)

**Figure 4:**
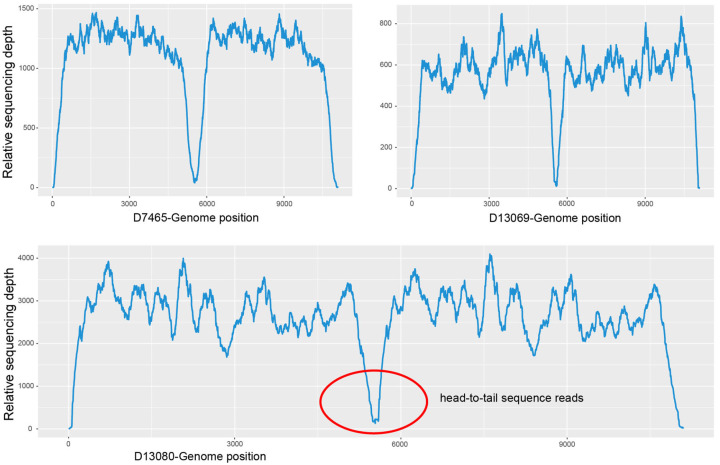
Relative sequencing depth of specimens positive (D7465, D13069, and D13080) for HBoV1 head-to-tail sequences. The concave part in the middle corresponds to the head-to-tail sequences, especially that in the red circle of D13080.

To validate the presence of the HBoV1 circular genome results in clinical specimens, there were three positive for HBoV1 head-to-tail sequences (D7465, D13069 and D13080) selected (Figure 4) for mNGS and two negative (D7133 and D13112) used as controls. No head-to-tail reads were detected in the negative specimens D7133 and D13112, while the head-to-tail reads were detected in all three positive specimens with the following read counts: D7465 with 44 reads, D13069 with 34 reads, and D13080 with 153 reads. The relative abundances of head-to-tail reads among all HBoV1 reads were 0.46‰, 0.12‰, and 0.75‰ for D7465, D13069, and D13080, respectively.

## 4. Discussion

In 2011, all possible head/tail primer combinations were designed by Lüsebrink et al. to search for the HBoV1 circular genome. Unexpectedly, only head-to-tail intermediates were detected in HBoV1 DNA positive specimens [19], rather than the expected head-to-head or tail-to-tail intermediates. Subsequently, the head-to-tail intermediates of HBoV 2-4 were detected in fecal specimens [20,21,22].

The members of the family Parvoviridae have single-strand linear DNA of about 5 kb. The coding sequence is flanked by short (116–385 bp) imperfect palindromes that can fold into hairpin structures. Parvovirus hairpins vary in size, sequence, and secondary structure among genera, but are quite conserved within a particular genus. All members of a genus are either homotelomeric or heterotelomeric, which markedly influences their biology. Individually, these telomeres give rise to viral replication origins in replicative form (RF) DNA, and together, they contain most of the cis-acting information required for both viral DNA replication and packaging [15]. The rolling hairpin replication model in parvoviruses results in head-to-tail and tail-to-tail intermediates. However, the rolling-circle replication produces head-to-tail intermediates. The starting point in many rolling circles is characterized by palindrome elements, which can switch between base pairing within or between chains. Therefore, the presence of head-to-tail sequences necessitates further study into the role the palindromic elements during replication.

To confirm the head-to-tail sequences, 16 specimens were selected for amplicon sequencing, and 60 head-to-tail sequences were harvested. These sequences were classified into 23 types according to the length of the head-to-tail sequences. Multiple head-to-tail sequences with different abundances can be detected in one specimen, while the same head-to-tail sequence can be detected in multiple specimens. Moreover, the 374nt head-to-tail sequence, a part of the 5384nt circular genome, showed an average abundance of 63.77% (variance σ^2^ = 0.04), which was followed by the 288nt head-to-tail sequence with an average abundance of 9.81% (variance σ^2^ = 0.007). All the head-to-tail sequences included the OriR, which is necessary for replication initiation. The data showed that these diverse head-to-tail sequences were present in clinical specimens and not as a result of artificial products of amplification and sequencing errors. The biological significance of the circular genome should be explored based on the structures retained in the head-to-tail sequences of HBoV2, which are important for DNA replication and virus assembly [23].

Although these head-to-tail sequences from amplicon sequencing were classified into 23 types according to the length of head-to-tail sequences, these sequences of the same length exhibited variants at the nucleotide level, as the 374nt type consisted of 374a and 374b in a ratio of 7.24:1. The secondary structures of these variants showed that the differences between two variants of one type occurred in the unpaired loop regions of the palindrome sequences, and these differences are complementary in reverse. This can be explained as the switch of the template chain in the process of replication, as reported in the rolling-circle “melting-pot” replication model [30]. For the circular genomes of porcine circovirus, both the complementary strand and the palindromic strand can serve as templates during the initiation and termination of DNA replication. There is no hydrogen bond between the positive chain and the negative chain to maintain any stable double helix conformation, but the four reverse–repeat chains are still close to each other and form a four-chain tertiary structure side by side. A rolling-circle “melting-pot” replication model was supposed, in which the replication protein (Rep) binds specific nucleotides and makes the loop and the palindromic sequences unstable without forming a cruciform structure. The Rep protein complex triggered an unstable field-melting pot that keeps all four chains of reverse–repeat sequences in a molten state. The rolling-circle melting-pot replication model allows for the “terminal repeat correction” of the left and right arms of OriR in the head-to-tail sequence, enabling the generation of wild-type palindromes or the formation of new palindromes in the progeny viruses [30]. 

However, there are several limitations of this research. First of all, although the diversity of the head-to-tail sequences, especially those shown in one specimen, was revealed in the study, the circularization mechanism and biological significance of these diverse head-to-tail sequences remain unknown, warranting further investigation. Secondly, the clinical significance of the head-to-tail sequences has not been explored, which will be done in the near future.

In conclusion, the head-to-tail sequences of HBoV1 detected from a large number of clinical specimens exhibited high diversity. They were classified into 32 types on the basis of TA cloning and Sanger sequencing from 42 specimens positive for head-to-tail sequences, and 23 types in length of head-to-tail sequences harvested by amplicon sequencing from 16 specimens positive for head-to-tail sequences. The circular genome sequences were confirmed by whole-genome mNGS. The secondary structure analysis of the head-to-tail sequences revealed the variants of one type might be generated by the switch of template chain in the melting-pot to get their complementary reverse sequences in the process of rolling-circle replication.

## Figures and Tables

**Table 1:** Primers designed for amplification of the head-to-tail sequences of HBoV1.

Round	Name	Position	Sequences (5′-3′)
First	HBoV1-F1-Tail (+)	JQ923422:5187-5206	gcttctgcttacaagttcct
	HBoV1-R1-Head (−)	JQ923422:365-346	ggaggattgaaagccatagt
Second	HBoV1-F2-Tail (+)	JQ923422:5283-5300	tggtgttaccgtctcgaa
	HBoV1-R2-Head (−)	JQ923422:272-254	aggaagtgcagcagcttaa

**Table 2:** Various linker sequences of the HBoV1 head-to-tail sequences compared to the 3′LEH and the 5′REH sequences of reference sequence LC756667.

Type	No. of Sequences	5′ REH Located on the Position of JQ923422	Linker Sequences		3′ LEH Located on the Position of JQ923422
			Sequences	Position on 5′ REH of JQ923422	
5	1	5283–5451	GCGCATGTTATGGATTACATCAT	unknown	196–272
9	14	5283–5402	GCTGATATAAAACT	5467–5480	119–272
11	4	5283–5402	GCTGATATAAAACT	5467–5480	119–272
13	6	5283–5426	ATGTACAACAACAACACATTAAAAGATAT	5491–5519	139–272
14	4	5283–5402	GCTGATATAAAACTAAGATGGCGCATGTAC	5467–5496	112–272
20	1	5283–5402	GCTGATATAAAACTAAGATG	5467–5486	73–272
21	1	5283–5402	GCTGATATAAAACTAAGATG	5467–5486	71–272
23	1	5283–5426	ATGTACAA	5491–5498	72–272
24	2	5283–5402	GCTGATATAAAACTAAGATGGCGCATGTACA	5467–5497	67–272
28	2	5283–5442	AAAGT	unknown	69–272
29	1	5283–5429	TAGATCA	unknown	56–272
31	1	5283–5444	GC	5467–5468	48–272
32	1	5283–5402	GCTGATATAAAACTAAGATGGCG	5467–5489	17–272

**Table 3:** Alignment results between different variants of the same type in length determined by amplicon sequencing and the reference sequence LC756667.

Variants	Alignment Results between Variants and Reference Sequence		
288a	1–120 5283–5402	121–144 5403–5426	145–288 129–272
288b	1–120 5283–5402	121–144 5420–5397 *	145–288 129–272
304a	1–120 5283–5402	121–149 5403–5431	150–304 118–272
304b	1–120 5283–5402	121–149 5420–5392 *	150–304 118–272
374a	1–124 5283–5406	125–242 23–140	243–374 141–272
374b	1–124 5283–5406	125–242 118–1 *	243–374 141–272

* The complementary reverse sequences corresponding to the reference sequence LC756667.

## Data Availability

The datasets presented in this study can be found in online repositories, and more datasets generated and analyzed during the current study are available from the corresponding author on reasonable request.
